# Retrogradation of Waxy Rice Starch Gel in the Vicinity of the Glass Transition Temperature

**DOI:** 10.1155/2013/549192

**Published:** 2013-06-06

**Authors:** Sanguansri Charoenrein, Sunsanee Udomrati

**Affiliations:** Department of Food Science and Technology, Faculty of Agro-Industry, Kasetsart University, Bangkok 10900, Thailand

## Abstract

The retrogradation rate of waxy rice starch gel was investigated during storage at temperatures in the vicinity of the glass transition temperature of a maximally concentrated system (*T*_*g*_′), as it was hypothesized that such temperatures might cause different effects on retrogradation. The *T*_*g*_′ value of fully gelatinized waxy rice starch gel with 50% water content and the enthalpy of melting retrograded amylopectin in the gels were investigated using differential scanning calorimetry. Starch gels were frozen to −30°C and stored at 4, 0, −3, −5, and −8°C for 5 days. The results indicated that the *T*_*g*_′ value of gelatinized starch gel annealed at −7°C for 15 min was −3.5°C. Waxy rice starch gels retrograded significantly when stored at 4°C with a decrease in the enthalpy of melting retrograded starch in samples stored for 5 days at −3, −5, and −8°C, respectively, perhaps due to the more rigid glass matrix and less molecular mobility facilitating starch chain recrystallization at temperatures below *T*_*g*_′. This suggests that retardation of retrogradation of waxy rice starch gel can be achieved at temperature below *T*_*g*_′.

## 1. Introduction

The retrogradation of starch has been defined as a process which occurs when the molecules comprising gelatinized starch begin to reassociate in an ordered structure [1]. Retrogradation can lead to an obvious increase in the firmness of stored baked goods [2] and frozen cooked rice [3], making them unattractive to consumers. However, in some products, retrogradation can provide a desirable quality such as in the manufacture of rice stick noodles [4], resistant starch type 3 [5] croutons, and bread crumb [6]. For this reason, numerous studies have been performed to examine the factors affecting the retrogradation of starch. Water content and storage temperatures play key roles in the extent of retrogradation. The maximum extent of retrogradation of most starches is attained in starch gels containing 50%–60% solids [7–10]. It was also found that starch gels retrograde faster when stored at 4−6°C [11, 12].

 Glass transition is a second-order phase transition that occurs over the temperature range at which amorphous solid materials (glassy materials) are transformed to a metastable leathery state [13]. A special glass transition temperature, denoted as *T*_*g*_′, has been defined as the glass transition of a maximally freeze-concentrated system [14]. *T*_*g*_′ plays a key role in the quality and storage stability of frozen products because the rate of deteriorative changes in frozen food is closely related to *T*_*g*_′ [13, 15]. Below *T*_*g*_′, where the food matrix is in a glassy state, molecular mobility decreases and consequently reduces the rate of deteriorative changes involving molecular mobility. Due to the fact that retrogradation of gelatinized starch involves the movement and rearrangement of starch chains to form a junction zone [16], we hypothesized that the extent of retrogradation process of starch gels at temperatures below and above *T*_*g*_′ should be different. Although there have been some studies on the influence of storage temperatures on the extent of retrogradation, these studies used ambient temperature, refrigeration (4 or 5°C), and frozen storage (−20°C) [11, 17] but not temperatures in the vicinity of *T*_*g*_′. Moreover, all of these experiments used samples which were gelatinized in differential scanning calorimeter (DSC) pans. This static gelatinization might not mimic the real heating process of starch suspension which usually includes stirring the suspension during application of heat. Heating with shear would completely paste the starch suspension while heating without shear might cause incomplete pasting. Moreover previous studies [10] have shown that maximum retrogradation occurred in rice starch gels with 40%–60% water content. In this paper, we present an alternative method to prepare completely gelatinized starch gels and then limit the water content to 50% to provide maximum retrogradation. This research will make a contribution toward an improvement in the acceleration or retardation of retrogradation of starch-based products. 

## 2. Materials and Methods

### 2.1. Materials

Waxy rice starch was made from Thai waxy rice grains (RD 6), grown in the Kalasin province area and aged at least six months.

### 2.2. Flour and Starch Preparation

Rice kernels were soaked in water for 4 h and then ground with water using a double-disc stone mill. The slurry was centrifuged for 15 min and dried at 45°C for 15 h. The flour was ground in a hammer mill and passed through a 100-mesh sieve; then it was stored at room temperature in sealed plastic bags. For the isolation of waxy rice starch from waxy rice flour, the method of Hogan [18] was used. Waxy rice flour was mixed with sodium hydroxide solution. The slurry was stirred, filtered, and centrifuged. The sediment was washed with water, neutralized with hydrochloric acid solution, and dried. The rice starch was then ground in a hammer mill and passed through a 100-mesh sieve. The granule size of was 2.8–5.1 *μ*m with mean diameter of 3.8 ± 0.7 *μ*m. The protein, fat, and ash contents of waxy rice starch were 0.20 ± 0.00, 0.05 ± 0.00, and 0.03 ± 0.00 g/100 g dried solid by the AACC Method 46-12, 30-25, and 08-01, respectively [19]. The moisture content was 11.07 ± 0.10 g/100 g by the AACC Method 44-15A [19]. The amylose content of waxy rice starch was 6.49 ± 0.13 g/100 g dried solid by the method of Juliano [20].

### 2.3. Waxy Rice Starch Gel Preparation

A starch suspension (10% w/w) was prepared by mixing the waxy rice starch in distilled water and stirring continuously at 250 rpm for 1 h followed by 200 rpm at 95°C for 1 h. The gelatinized starch sample was then put in stainless steel DSC pans. Water in each sample was allowed to evaporate at room temperature until the final water content was 50% as determined by weighing. The final sample weight in each pan was 20–30 mg. The pans were hermetically sealed to prevent moisture loss. The sealed pans were separated into two sets with one set being used for glass transition determination and the other set being used in the retrogradation study.

### 2.4. Glass Transition Temperature (*T*_*g*_′) Determination

A Pyris-1 DSC (Perkin Elmer, Norwalk, CT, USA) equipped with an intracooler subambient accessory was used. Nitrogen gas was used as the purge gas at a flow rate of 20 mL/min during calibration and measurements. The instrument was calibrated using indium and ice. An empty pan was used as a reference sample. Each sample in a sealed DSC pan was cooled to −60°C and then heated to 25°C at 5°C/min to determine the glass transition temperature of a nonannealed state. For the isothermal annealing treatment, the samples were cooled to −60°C and held for 15 min, heated to three different annealing temperatures (−4, −7, and −10°C) in the vicinity of the *T*_*g*_′ of a nonannealed sample and held at this temperature for 15 min, and cooled back to −60°C at 10°C/min and reheated to 25°C at 5°C/min to locate the *T*_*g*_′. The *T*_*g*_′ was indicated by an onset temperature of the heat capacity change, which was determined using the computer software program associated with the Perkin Elmer instrument. All measurements were run in triplicates.

### 2.5. Amylopectin Retrogradation Analysis

The sealed DSC pans with gelatinized starch were frozen to −30°C in a cooling bath and held for 1 h and then immediately stored at −8, −5, −3, 0, and 4°C for 5 days. Storage at −8, −5, and −3°C was done in a cooling bath while the samples at 0 and 4°C were kept in a low temperature incubator (Model IPP 400, Memmert, Germany). After storage, the pans were left to stand for 30 min at room temperature to equilibrate and then heated from 25 to 120°C in the DSC at 10°C/min to observe the melting peak of the retrograded starch gels. All measurements were performed in triplicate.

## 3. Results and Discussion

### 3.1. Glass Transition

**Figure 1:**
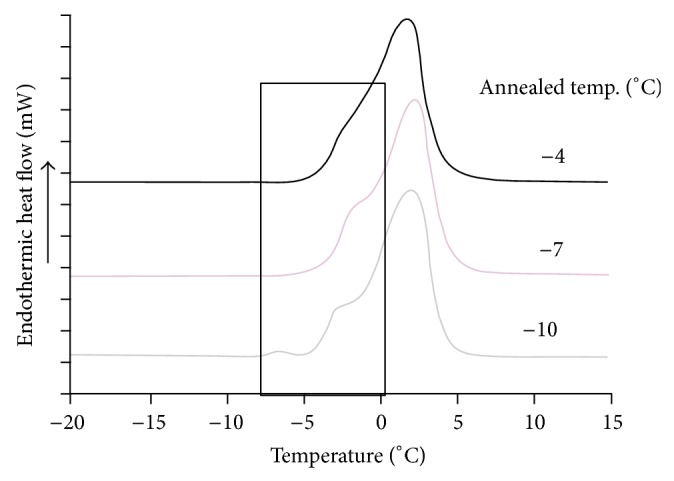
Differential scanning calorimetry thermograms showing glass transition temperature (*T*_*g*_′) of waxy rice starch gel samples which were isothermally annealed at −4, −7, and −10°C for 15 min.

In a system that is allowed to form the maximum amount of ice, the glass transition of this maximally freeze-concentrated matrix occurs at *T*_*g*_′ and is independent of the initial solids fraction (before freezing) [21]. However, if the maximum amount of ice is not formed in the system, the resulting unfrozen matrix will be more dilute. Annealing is a way to form a maximally freeze-concentrated phase. The DSC thermograms showing the *T*_*g*_′ values of the gelatinized waxy rice starch isothermally annealed at different temperatures are presented in Figure 1. The *T*_*g*_′ value of the gelatinized waxy rice starch was about −5°C in the nonannealed state. At the three different annealing temperatures of −4, −7, and −10° C, the *T*_*g*_′ value of gelatinized waxy rice starch was −4, −3.5, and −4.2°C, respectively. The annealing temperature of −7°C resulted in the highest *T*_*g*_′ value and the most clearly detectable among the three annealing temperatures. This might have occurred because this temperature, which was slightly below the *T*_*g*_′ of the gelatinized waxy rice starch (−5°C), was high enough to have sufficient molecular mobility for ice formation and yet also low enough to maintain the amorphous glass matrix as discussed by Lim et al. [22]. Our *T*_*g*_′ result of −3.5°C agreed relatively well with Slade and Levine [23], Roos and Karel [24], and Israkarn and Charoenrein [25] who found that the *T*_*g*_′ values of gelatinized wheat starch, gelatinized waxy corn starch, and cooked rice stick noodles were −5, −6, and −4°C, respectively.

### 3.2. Amylopectin Retrogradation

**Figure 2:**
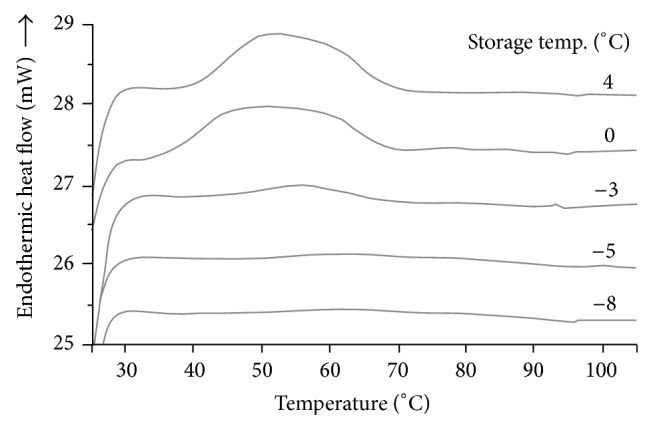
Differential scanning calorimetry melting endotherms obtained from waxy rice starch gel samples containing 50% water content stored at 4, 0, −3, −5, and −8°C for 5 days.

**Figure 3:**
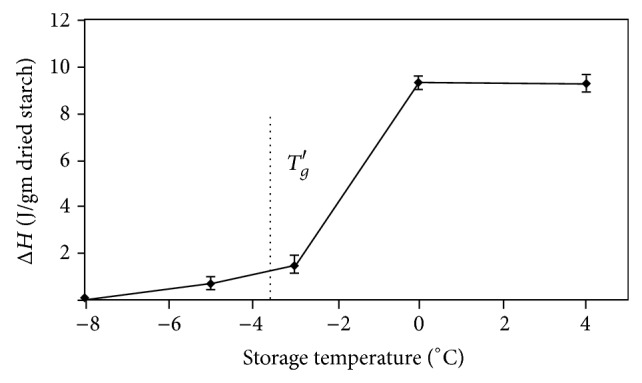
Enthalpy of melting retrograded waxy rice starch gel samples containing 50% water content as a function of storage temperature after 5-day storage.

In this study, waxy rice starch was selected because it showed a well-defined melting peak of retrograded amylopectin at temperature range of 40–75°C. The method of gel preparation used in this study included (1) shearing during heating, which provided a fully gelatinized starch sample, and (2) control of the water content at 50% to obtain the maximum retrogradation extent. Gelatinized waxy rice starch samples stored at various temperatures in the vicinity of the *T*_*g*_′ for 5 days showed differences in enthalpy of melting of the retrograded starch gels (Figures 2 and 3). The peak of melting of retrograded starch was large in samples stored at 4 and 0°C. Samples stored at −3°C showed a small peak while a very small peak and no peak were shown in samples stored at −5 and −8°C, respectively, which were temperatures below *T*_*g*_′ (at −3.5°C). This indicated that at temperature below the glass transition temperature, the movement of starch chains to form junction zone of retrogradation was hindered. The results also agreed with Baik et al. [26] who reported that waxy rice starch gels stored at subzero temperature (−12°C) showed a lower degree of recrystallization of starch than those stored at 4°C. However, their intervals of storage temperature were greater than those in our studies.

## 4. Conclusions

The onset of *T*_*g*_′ of the gelatinized waxy rice starch gel annealed at −7°C for 15 min was –3.5°C. The results showed that rice starch gels retrograded substantially when stored at 4°C for 5 days. Decreases in the enthalpy of melting of retrograded starch gel were observed in samples stored at –3, −5, and −8°C. This was due to the more rigid glass matrix and less molecular mobility at temperatures below *T*_*g*_′. These results suggested that the retardation of retrogradation of waxy rice starch gel can be manipulated by a temperature below *T*_*g*_′ and acceleration could be carried out at temperature above *T*_*g*_′.
